# Psychosocial morbidity in women with abnormal cervical cytology managed by cytological surveillance or initial colposcopy: longitudinal analysis from the TOMBOLA randomised trial

**DOI:** 10.1002/pon.4163

**Published:** 2016-06-14

**Authors:** S. Fielding, K. Rothnie, N. M. Gray, J. Little, M. E. Cruickshank, K. Neal, L. G. Walker, D. Whynes, S. C. Cotton, L. Sharp, In association with the TOMBOLA group

**Affiliations:** ^1^ Medical Statistics Team, Division of Applied Health SciencesUniversity of AberdeenAberdeenScotland; ^2^ Faculty of Epidemiology and Population Health, London School of Hygiene and Tropical Medicine and Faculty of MedicineImperial College LondonLondonUK; ^3^ Scottish Improvement Science Collaborating Centre, School of Nursing and Health SciencesUniversity of DundeeDundeeScotland; ^4^ School of Epidemiology, Public Health and Preventive MedicineUniversity of OttawaOttawaCanada; ^5^ Division of Medical and Dental EducationUniversity of AberdeenAberdeenScotland; ^6^ Consultant EpidemiologistLondon and South East PHE CentresLondonUK; ^7^ Medical Research CentreUniversity of HullHullUK; ^8^ School of EconomicsUniversity of NottinghamNottinghamUK; ^9^ Division of Applied Health SciencesUniversity of AberdeenAberdeenUK; ^10^ Institute of Health & SocietyNewcastle UniversityNewcastleUK

**Keywords:** cancer, oncology, colposcopy, cytology, psychosocial morbidity

## Abstract

**Objective:**

To compare psychosocial outcomes (follow‐up related worries and satisfaction with follow‐up related information and support) over 30 months of two alternative management policies for women with low‐grade abnormal cervical cytology.

**Methods:**

Women aged 20–59 years with low‐grade cytological abnormalities detected in the National Health Service Cervical Screening Programme were randomised to cytological surveillance or initial colposcopy. A total of 3399 women who completed psychosocial questionnaires at recruitment were invited to complete questionnaires at 12, 18, 24 and 30 months. Linear mixed models were used to investigate differences between arms in the two psychosocial outcomes. Each outcome had a maximum score of 100, and higher scores represented higher psychosocial morbidity.

**Results:**

On average, over 30 months, women randomised to colposcopy scored 2.5 points (95%CI −3.6 to −1.3) lower for follow‐up related worries than women randomised to cytological surveillance. Women in the colposcopy arm also scored significantly lower for follow‐up related satisfaction with information and support (−2.4; −3.3 to −1.4) over 30 months. For both outcomes, the average difference between arms was greatest at 12th‐ and 18th‐month time points. These differences remained when the analysis was stratified by post‐school education.

**Conclusions:**

Women with low‐grade cytology, irrespective of their management, have substantial initial psychosocial morbidity that reduces over time. Implementation of newer screening strategies, which include surveillance, such as primary HPV screening, need to consider the information and support provided to women. © 2016 The Authors. *Psycho‐Oncology* published by John Wiley & Sons Ltd.

## Introduction

Many women have an abnormal cervical screening test, based on cytological and/or human papilloma virus (HPV) testing. Irrespective of the nature of the initial test, abnormal screens require further investigation. A range of options exist, the main ones being repeat testing (by cytology and/or HPV) and colposcopy examination. Both are recognised as acceptable in a range of guidelines internationally 1, 2, 3, 4.

Like all screenings, cervical screening involves a balance between harms and benefits. Harms include adverse psychosocial sequelae identified across the entire screening process from initial screening, investigation and potentially beyond 5. Psychosocial distress associated with alternative management policies has been little investigated. The debate about relative levels of anxiety between repeat testing (i.e*.* surveillance) and colposcopy has been ongoing for at least two decades 6, 7, but there are only two randomised trials of women with low‐grade cytology. The first found no difference at 12 months in mean distress or anxiety scores (assessed using the General Health Questionnaire and the Spielberger State Trait Anxiety Inventory) between those women randomised to a repeat cytology test in 6 months or a choice between repeat cytology and an immediate colposcopy 8. The second trial (known as TOMBOLA) found no difference in anxiety or depression (assessed using the Hospital Anxiety and Depression Scale (HADS)) over 30 months of follow‐up between women randomised to cytological surveillance or initial colposcopy, although anxiety and depression were significantly lower in women in the colposcopy arm at 6 weeks 9.

A potential limitation of these reports is they considered generalised measures of psychosocial morbidity. Women attending for cervical screening report a range of specific concerns, including worries about cervical cancer, fertility, psychosexual issues and body image, and it is likely that generalised measures of anxiety and depression do not capture these adequately 10. Furthermore, it is possible that, in the same women, patterns of generalised anxiety and patterns of these more specific concerns may differ. To counter this, generalised and disease‐specific instruments could be used in combination to assess the full range of aspects of health relevant to the population concerned.

In current evidence, it is not clear whether the psychosocial morbidity associated with different management policies differs between subgroups of women. There is some evidence, albeit limited, that the psychosocial sequelae of abnormal screening tests and follow‐up may be more pronounced in women of lower socio‐economic status or with poorer health literacy 11, 12, 13. Drolet found that among women with abnormal smear results, lower socio‐economic level was significantly associated with having clinically meaningful anxiety at 12 weeks 11. In women referred for colposcopy, Orbell found that those residents in areas of higher social deprivation reported significantly higher anxiety scores 12, while Sharp found that low health literacy was significantly related to higher levels of distress 13. No studies appear to have investigated whether the psychological impact of different management policies differs according to women's socio‐economic status or health literacy.

Using data from the TOMBOLA trial, the primary aim here was to compare the psychosocial morbidity (worries and satisfaction with information and support) over 30 months of follow‐up in women managed by the two alternative policies (cytological surveillance versus initial colposcopy). Data were available for 3399 women. The secondary aim was to investigate whether these psychosocial outcomes differed in women with different levels of post‐school education, a marker of both socio‐economic status 14 and health literacy 15.

## Methods

### Participants and recruitment

The TOMBOLA trial design and sample size calculation have been previously described 16. In brief, women resident in Grampian, Tayside or Nottingham, aged 20–59 years with a routine cervical screening test showing low‐grade abnormalities (borderline nuclear abnormalties (BNA) or mild dyskaryosis) within the National Health Service Cervical Screening Programmes between October 1999 and October 2002 were recruited. Eligible women could have had up to one additional BNA result in the previous 3 years. Women with previous treatment for proven or suspected lesions or who were pregnant were not eligible. Women were randomised in equal proportions to either cytological surveillance or initial colposcopy, using a telephone randomisation service provided by the University of Aberdeen. All women were followed up for 36 months, at which point they were invited to attend for an exit examination, including colposcopy.

### Management policies

Cytological surveillance consisted of repeat cytology tests every 6 months in primary care. Women returned to routine recall if they had three consecutive negative results. Women with a cytology test showing moderate dyskaryosis or worse or three inadequate tests were referred for colposcopy and managed according to local protocols. Otherwise, women continued to receive six monthly cytology tests. Women randomised to initial colposcopy received an appointment to attend hospital for colposcopy examination and were further randomised to immediate treatment by large loop incision of the transformation zone or 2–4 targeted punch biopsies and selective recall for treatment. If an abnormal transformation zone was seen at colposcopy, women received the intervention assigned in this second randomisation. They were subsequently followed up every 6 months with cytology tests in primary care. If the transformation zone was normal, no additional procedures were carried out at colposcopy, and women were followed by annual cytology tests in primary care. After three consecutive normal smears, women were returned to management by routine recall. Cytological results were monitored with subsequent management (i.e. subsequent test date or referral to colposcopy) based on these. Women re‐referred to colposcopy during follow‐up attended local National Health Service clinics where they were treated, if required, according to local protocols.

### Data collection

At recruitment, participants completed socio‐demographic and psychosocial questionnaires. Women were invited to complete psychosocial questionnaires by post at 12, 18, 24 and 30 months post‐recruitment. The psychosocial questionnaire included HADS 17 and the process outcome specific measure (POSM). The POSM was developed within TOMBOLA and includes 14 questions covering a range of issues identified as important by women with an abnormal smear who were undergoing follow‐up, including concerns about cervical cancer, fertility and satisfaction with information and support 10. In brief, the questions in the recruitment questionnaire related to the time since receiving the cytology result, and those in the follow‐up questionnaires related to the previous 4 weeks. Response options were in the form of 5–7 level Likert scales. This instrument has been shown to have acceptable psychometric properties and good discriminant validity against the HADS 10, 18.

### Outcomes

The two outcomes of interest were derived from seven ‘core’ questions on the POSM 18. A previously published factor analysis 18 identified two constructs (Table S1): follow‐up related worries (four questions relating to worries about cervical cancer, general health, the result of the next cytology test and having sex) and satisfaction with follow‐up related information and support (three questions relating to feeling well enough informed, being satisfied with support from other people and how the woman felt about herself). An exploratory factor analysis was undertaken with varimax rotation, and the two factors explained 54.7% of the variance. Internal consistency of the resulting factors was assessed using Cronbach's alpha and was 0.769 for worries and 0.482 for information and support. More details can be found in the previous publication 18.

Item responses for each question were standardised to a score out of 100 (to account for a different number of response options) 18. For each construct, the standardised item scores for the relevant questions were averaged and standardised to a score out of 100. A higher score indicates more worries or greater dissatisfaction with information and support. To calculate scores for each construct, women had to answer all questions which formed that construct.

### Statistical methods

Baseline characteristics were summarised for each management arm using mean and standard deviation (SD) for continuous variables and number and percentage for categorical variables. The focus was to determine whether, on average, over the entire follow‐up period, the psychosocial morbidity differed between management arms. A subsidiary interest was to determine whether there were differences between arms in the pattern, or profile of the psychosocial morbidity during follow‐up, and if so, at which individual follow‐up time‐point differences were most evident. Therefore, each longitudinal outcome (at follow‐up time points 12, 18, 24, 30 months) was analysed using a linear mixed effects model (with unstructured covariance) to allow for the correlation in repeated measures and allow all women who had responded to at least one of the follow‐up assessments to be included 19. This analysis approach assumes data are missing at random, which was a plausible assumption in this setting. Models were adjusted for minimisation variables (age group, eligible smear, HPV status and trial centre) 20, baseline (recruitment) score for worries or information and support (as relevant) and depression at recruitment as measured by the HADS because this differed significantly between arms 9. Fixed effects for follow‐up time point, management arm, and an interaction between follow‐up time point and management arm were included; this interaction term tested whether there were different profiles of psychosocial morbidity over the entire follow‐up period between the two management arms. Models included random effects for participants and interaction between participants and follow‐up time point to allow for random intercepts and slope. All analyses were undertaken in sas v9.3 (SAS Institute, Cary, NC, USA). The models were run initially for all women (primary analysis) and then stratified by post‐school education/training (secondary analysis).

## Results

### Characteristics of participants

There were 3399 women eligible to complete the psychosocial questionnaires, of whom 1703 (50.1%) were randomised to cytological surveillance and 1696 (49.9%) to initial colposcopy (Figure S1). The management arms were well balanced (Table S2) in terms of women's socio‐demographic and clinical characteristics. Slightly over 40% of women were aged 20–29, one quarter were aged 30–39, one‐fifth were aged 40–49, and less than 10% were aged 50–59 years. Just over one quarter were recruited on the basis of a cytology test showing mild dyskaryosis. Less than 10% had another BNA cytology test in the previous 3 years. Ninety‐five per cent of women described their ethnic group as ‘white’. Just over a quarter had received no post‐school education, one‐fifth had received training through work, almost 30% had obtained a qualification other than a degree from college or university and just under one quarter had a degree.

### Psychosocial morbidity over time

**Figure 1:**
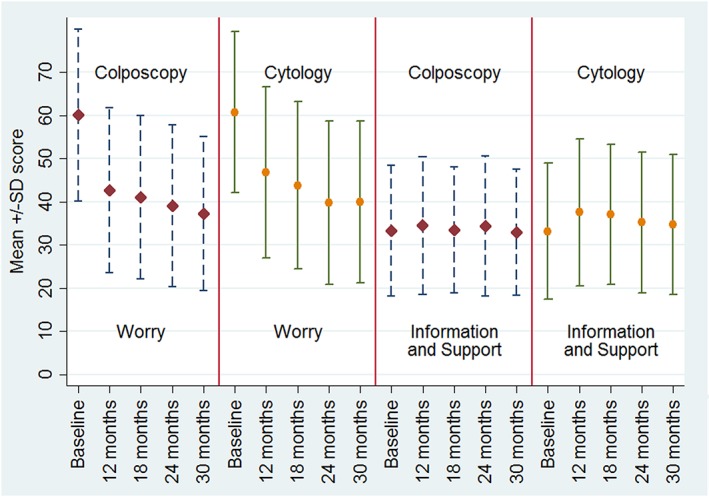
Mean scores^1^ for worries and satisfaction with information and support at recruitment and during follow‐up (split by treatment group).

Figure 1 shows mean (+/− standard deviation) scores for each of the outcome variables at each time point separately for the two management arms. The mean (SD) worries score declined from 60.1 (19.9) at baseline to 42.6 (19.1) at 12 months in the colposcopy arm, and by 30 months, it had fallen to 37.3 (17.8). The cytology arm at baseline was similar, with mean (SD) 60.7 (18.6), but by 30 months, was 40 (18.7). The mean satisfaction with information and support score changed little over time and was 33.3 (15.2) with a slight rise to 34.5 (15.9) at 12 months but returned to around 33 by 30 months in the colposcopy arm. In the cytology arm at baseline, the mean (SD) was 33.2 (15.7) rising to 37.6 (17.0) at 12 months and dropping back down to 34.7 (16.2) by 30 months.

1 Maximum score for each outcome is 100; higher scores indicate more worries or less satisfaction with information and support.

### Follow‐up related psychosocial morbidity: comparison of trial arms

Table 1 shows the results of the analysis comparing trial arms overall and at each time point. Over the entire observation period (12–30 months), the follow‐up related worries score in the colposcopy arm was, on average, 2.47 (95% CI −3.63 to −1.30) points lower than in the cytology arm over the entire follow‐up period, and this was statistically significant (*p* < 0.001). The pattern of follow‐up related worries differed significantly between arms (*p*‐value for interaction = 0.013). A difference in the worries score was evident at all time points; it was most pronounced at 12 months and least pronounced (and not significant) at 24 months.

**Table 1:** Mean (SD) observed scores for each outcome at each follow‐up time point and mixed effects model results for the differences between colposcopy and cytology: primary analysis

	Cytological surveillance		Initial colposcopy		Colposcopy minus cytology^a^			Time point‐management arm interaction *p*‐value
	*n*	Observed mean (SD)	*n*	Observed mean (SD)	Estimate	95% CI	*p*‐value	
Follow‐up related worries (*n* = 3198)								0.013
Across all time points	3198				−2.47	(−3.63, −1.30)	<0.001	
At individual time points								
12 months	1119	46.8 (19.8)	1147	42.6 (19.1)	−3.69	(−5.14, −2.25)	<0.001	
18 months	997	43.8 (19.4)	1050	41.0 (18.9)	−2.52	(−4.03, −1.01)	0.001	
24 months	915	39.8 (18.9)	965	39.0 (18.7)	−1.13	(−2.62, 0.36)	0.137	
30 months	859	40.0 (18.7)	923	37.3 (15.2)	−2.53	(−4.08, −0.99)	0.001	
Satisfaction with follow‐up related information and support (*n* = 3199)								0.013
Across all time points	3199				−2.35	(−3.32, −1.38)	<0.001	
At individual time points								
12 months	1113	37.6 (17.0)	1111	34.5 (15.9)	−3.14	(−4.46, −1.83)	<0.001	
18 months	994	37.0 (16.2)	1034	33.5 (14.6)	−3.38	(−4.67, −2.08)	<0.001	
24 months	936	35.2 (16.3)	986	34.4 (16.2)	−1.13	(−2.52, 0.27)	0.114	
30 months	861	34.7 (16.2)	916	33.0 (14.6)	−1.76	(−3.15, −0.67)	0.013	

a All models adjusted for age group, eligible smear, HPV status, trial centre, baseline depression and baseline score

CI, confidence interval; SD, HPV, human papilloma virus; standard deviation.

Over the entire observation period, satisfaction with follow‐up related information and support scores were, on average, 2.35 (95% CI −3.32 to −1.38) points lower in the colposcopy arm compared with the cytology arm (*p* < 0.001 for interaction). The interaction between time point and management was significant (*p* = 0.013) indicating that the profile of scores was different between arms. Satisfaction with information and support scores were lower (indicating greater satisfaction) at every time point in those randomised to colposcopy compared with those randomised to cytological surveillance, but at 24 months, the difference was not statistically significant.

### Follow‐up related psychosocial morbidity, by post‐school education

Figure S2 shows mean overall follow‐up related worries (Figure S2A) and satisfaction with information and support (Figure S2B) scores at each follow‐up time point for women in the four post‐school education categories. The pattern of worries scores across follow‐up was very similar for the four post‐school education groups. However, satisfaction with information and support scores did differ across the four education groups, with lowest scores (i.e. greatest satisfaction) obtained for those with no post‐school education.

Table S3 shows for each outcome, the average difference in scores over the entire observation period, between trial arms, stratified by post‐school education. For follow‐up related worries, in all four post‐school education groups, women in the colposcopy arm scored lower, but the difference was only statistically significant in women with qualifications through work (average difference 4.39 points, *p* = 0.002) and women with a degree (average difference 2.36 points, *p* = 0.031). Similarly, for satisfaction with follow‐up related information and support, women in the colposcopy arm were more satisfied than those in the cytological surveillance arm in all four subgroups. These differences reached statistical significance for those with no post‐school education (*p* = 0.005), those with qualifications through work (*p* = 0.016) and those with a degree (0.023).

## Discussion

The major finding was that follow‐up psychosocial morbidity was somewhat lower for women randomised to initial colposcopy than to cytological surveillance. This was seen consistently for both outcomes (follow‐up related worries and satisfaction with follow‐up related information and support).

For each outcome, the average difference in scores between arms was 2 points or less. Given that the maximum score a woman could attain was 100, this difference is clearly modest. Further work is needed to determine: whether a difference of this magnitude would represent a clinically meaningful difference in psychosocial well‐being/morbidity at either the individual or the population level; and whether any differences could translate into further disbenefits of screening such as non‐adherence to treatment, further follow‐up, or subsequent rounds of primary screening, which may be associated with the development of precancerous or cancerous lesions.

Some literature suggests that women may prefer more ‘active’ strategies for the follow‐up of abnormal cytology (such as colposcopy) to ongoing surveillance, but this is not consistent, and a range of factors influence these preferences, including cytology grade 8, 21, 22, 23, 24. In TOMBOLA, when women were asked (at the end of their follow‐up) about their satisfaction and preferences, although the majority were content with the management they had received, a minority would have preferred the alternative option, and most of these would have preferred colposcopy 25. The results of the current study could provide an explanation for why this is the case (i.e. because of a perception that it would reduce worries).

A range of explanations are possible for why the psychosocial morbidity is lower in the colposcopy arm. It has been suggested that the reasons for women preferring active follow‐up are that ‘treatment’ is performed more rapidly and that it provides a more definitive result 26. Together, these may provide women with resolution of the psychosocial uncertainties associated with receipt of an abnormal cytology result. While women in our study managed by initial colposcopy did receive colposcopy earlier than the first surveillance smear (due at 6 months), they may not have considered it to provide a definitive result because they were subsequently managed by 6 or 12 monthly cytology tests. A previous study has shown that, among women with high‐grade abnormal cytology, those who received treatment had a greater decline in anxiety compared with untreated women 11 suggesting the possibility that the lower psychosocial morbidity in the colposcopy arm could be because some women were treated. Another possible explanation is that, at colposcopy and any subsequent treatment appointments, women would have seen either a nurse colposcopist or a gynaecologist. It is possible that seeing a ‘specialist’ is more reassuring than seeing a general practitioner or nurse in primary care, because attention by a specialist promotes less distress because it is felt something is being done 27. Colposcopy is a more invasive procedure, and the process of examination and the possibility of viewing their examination on a TV screen may provide women with more reassurance than simply having a cytology sample taken. Furthermore, the written and verbal information provided by a specialist, or the opportunity to ask a specialist questions, may be more effective in resolving any uncertainties associated with abnormal cytology and its follow‐up. An individual's coping skills and coping styles influence psychological reactions to colposcopy 28 and have implications for the framing of information about management of abnormal cytology 29. Although we did not assess coping styles, it seems unlikely that our findings could be explained by differences between arms in the distribution of women's coping skills and styles; the randomised design and large sample size mean that these factors are most likely balanced across arms.

Although our primary interest was to consider the entire follow‐up period (12–30 months), in subsidiary analyses, we investigated whether there were differences between the arms at individual time points. For all outcomes, the differences between the arms were largest at the 12‐ and 18‐month time points, which is consistent with the pattern of women's self‐reported health during follow‐up 25. It is also consistent with other work suggesting that the quality of life impact of screening‐related events is generally fairly short‐term 30. It was noteworthy that for all three outcomes, there was no significant difference between the arms at 24 months; although the average score was still lower in the colposcopy arm. A possible explanation is that by 24 months, some women in the cytology arm may have been returned to routine recall, and this ‘resolution’ may have resulted in lower scores in that arm at that time point. However, if this explanation holds, we might also expect to see no difference in scores at the 30‐month time point, but in fact, differences between arms were again apparent (although smaller than at 12 and 18 months).

With the exception of satisfaction with information and support in women who had obtained qualifications through work, we found no evidence that the psychosocial morbidity scores during follow‐up differed in women with different levels of post‐school education (a marker of socio‐economic status and health literacy). In contrast, Orbell *et al*., found that lower socio‐economic status was correlated with higher anxiety in women who had colposcopy 12; however, anxiety was assessed 7 days after colposcopy, and socio‐economic status was based on an area‐based measure of deprivation; this is in contrast with the long‐term assessment and individual level measure of socio‐economic status in our study. Moreover, we found little evidence of a differential effect of initial colposcopy and cytological surveillance in relation to post‐school education. Across all these outcomes in all four post‐school education groups, women randomised to colposcopy consistently scored lower on average than women randomised to cytological surveillance, although this difference was not always statistically significant. This suggests that if choosing between a follow‐up policy of initial colposcopy or cytological surveillance, decision makers need not be overly concerned that one policy will differentially adversely affect particular socio‐economic groups.

### Strengths/limitations

The major strengths of this randomised controlled trial are its size, population basis, length of follow‐up and longitudinal assessment of outcomes. It is the first randomised study to have compared the psychosocial morbidity in women managed by colposcopy with surveillance. Although we addressed a range of issues identified by women undergoing follow‐up, women may have other specific concerns and worries that were not considered here, for example, future fertility or body image 31, 32, 33. The outcomes (worries and satisfaction with information and support) were derived from factor analysis. While the reliability was good for the worries factor (C_α_ = 0.769), it was poorer for the satisfaction with information and support factor (C_α_ = 0.482) 18. Although women were recruited to the trial some time ago and primary HPV testing is being implemented or considered in many programmes 3, women with positive HPV tests will still require triage or follow‐up. For women not at sufficient risk for immediate colposcopy, options include repeat cytology and/or HPV testing at 6 or 12 months; thus, our findings remain relevant. In the sentinel sites in England, cytology negative/high risk HPV positive women will have repeat HPV testing after 12 months 34. Since TOMBOLA was conducted, the written information provided to women is more standardised, and there has been an explosion of other sources of information, for example, on the internet. The likely impact of this on women's psychosocial well‐being is unknown.

## Conclusions

Women with low‐grade cytology managed by colposcopy compared with cytological surveillance have, on average, a lower psychosocial morbidity during follow‐up. Although the difference is modest, implementation of newer screening strategies, which incorporate a surveillance pathway such as primary HPV screening, needs to consider the information and support provided to women.

## Ethics approval

Ethical approval was obtained from the Joint Research Ethics Committee of NHS Grampian and the University of Aberdeen (Reference 970072), the Tayside Committee on Medical Research Ethics (170/99) and the Nottingham Research Ethics Committee (PA129701).

## Conflict of Interest

The authors have declared that there is no conflict of interest.

## Supporting information


**Figure S1.** Flowchart of recruitment, randomisation and completion of questionnaires
**Figure S2A:** Worries
**Figure S2B:** Information and support
**Table S1:** Question stems
**Table S2:** Baseline characteristics of TOMBOLA participants eligible to be included in psychosocial analysis
**Table S3:** Mixed effects model results for the differences between colposcopy and cytology across all time points: secondary analysis (stratified by post‐;school education)

Supporting info item

Supporting info item

Supporting info item
