# Supplementary material for: Psychosocial morbidity in women with abnormal cervical cytology managed by cytological surveillance or initial colposcopy: longitudinal analysis from the TOMBOLA randomised trial
Source: Psychooncology. 2016 Jun 14;26(4):476–83. doi: 10.1002/pon.4163 (PMC5412834; doi:10.1002/pon.4163)
Supplement: Supplementary file 1 — Supporting info item [file PON-26-476-s001.docx]

###### Figure S1. Flowchart of recruitment, randomisation and completion of questionnaires

**9480 ineligible, or did not attend for recruitment, or did not consent**

**13956 women assessed for eligibility**

**4476** **randomised**

**37 randomised but ineligible**

**4439** **randomised and confirmed to be eligible**

**2216 randomised to initial colposcopy**

**2223 randomised to cytological surveillance**

**520 recruited before PS^1^ questionnaires introduced**

**1696 recruited after PS^1^ questionnaires introduced**

**520 recruited before PS^1^ questionnaires introduced**

**1703 recruited after PS^1^ questionnaires introduced**

3399 included in PS analysis here

^1^ PS=psychosocial
