# Supplementary material for: Psychosocial morbidity in women with abnormal cervical cytology managed by cytological surveillance or initial colposcopy: longitudinal analysis from the TOMBOLA randomised trial
Source: Psychooncology. 2016 Jun 14;26(4):476–83. doi: 10.1002/pon.4163 (PMC5412834; doi:10.1002/pon.4163)
Supplement: Supplementary file 2 — Supporting info item [file PON-26-476-s002.docx]

***Figure S2. Mean scores^1^ for worries and satisfaction with information and support at recruitment and during follow-up, for women in the four post-school education categories.***

***Figure S2A. Worries***


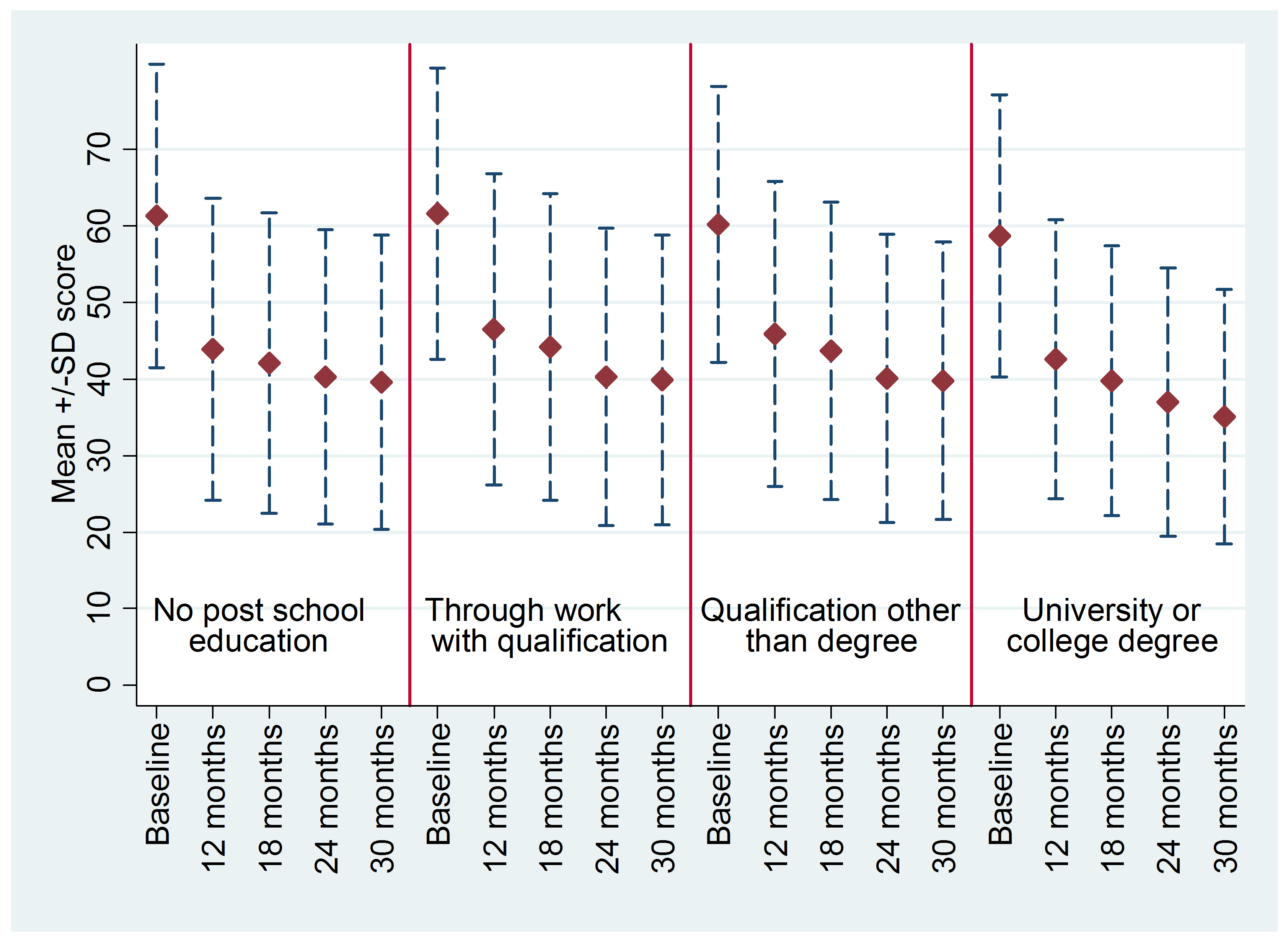


***Figure S2B. Information and support***


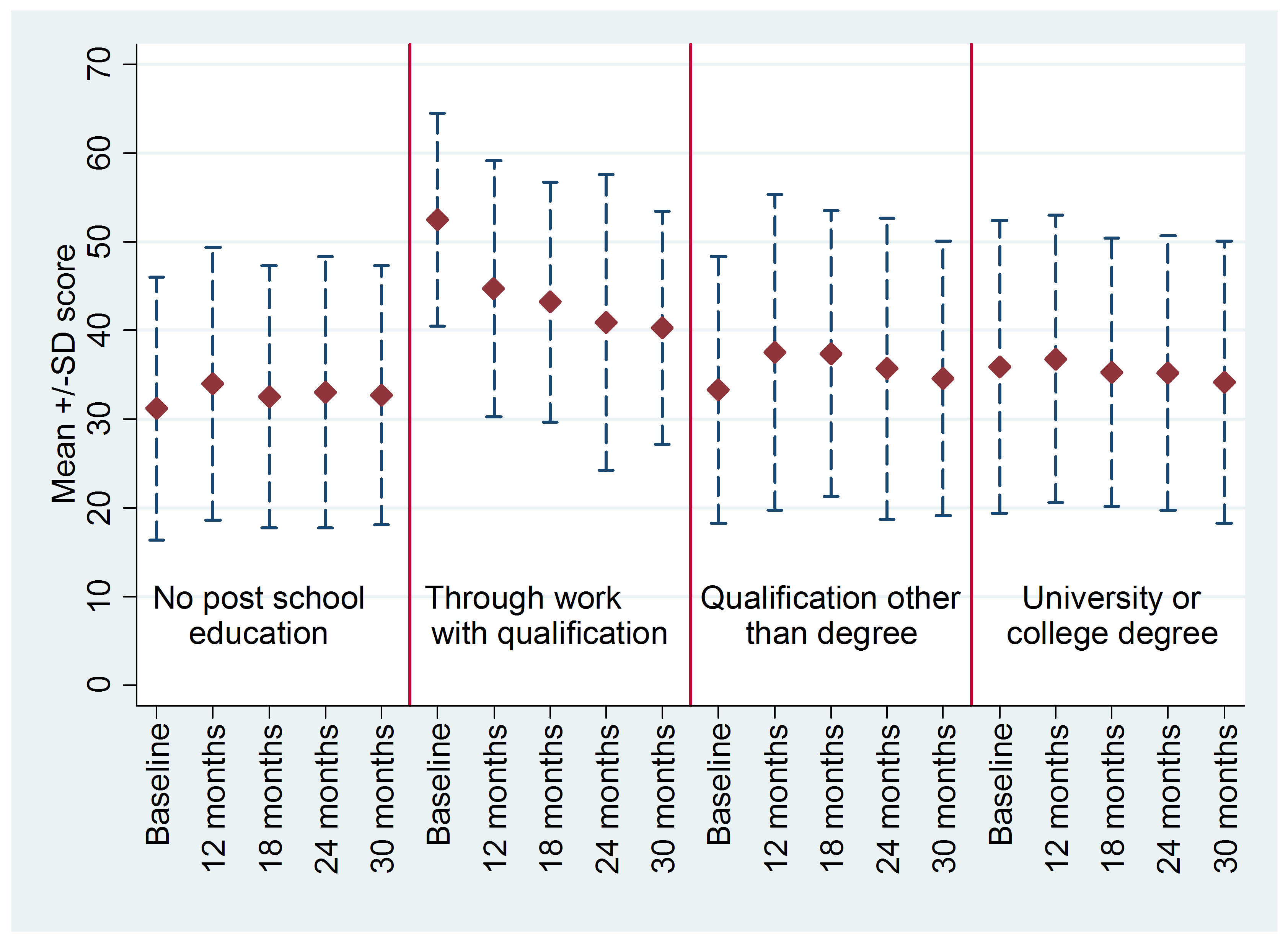


^1^ Maximum score for each outcome is 100; higher scores indicate more worries, or less satisfaction with information and support
