# Supplementary material for: Psychosocial morbidity in women with abnormal cervical cytology managed by cytological surveillance or initial colposcopy: longitudinal analysis from the TOMBOLA randomised trial
Source: Psychooncology. 2016 Jun 14;26(4):476–83. doi: 10.1002/pon.4163 (PMC5412834; doi:10.1002/pon.4163)
Supplement: Supplementary file 3 — Supporting info item [file PON-26-476-s003.docx]

**Table S1: Question stems**

| ***Question stem*** | ***Factor*** |
| --- | --- |
| In the last month I have been worried that I may have cervical cancer.^1^ | follow-up related worries |
| In the last month I have been worried about my general health.^1^ | follow-up related worries |
| In the last month I have been worried that my next smear will show changes to the cells. ^1^ | follow-up related worries |
| In the last month I have been worried about having sex.^1^ | follow-up related worries |
| In general I feel well enough informed about my follow-up.^1^ | satisfaction with follow-up related information and support |
| In the last month I have generally been satisfied with the support I have had from other people.^1^ | satisfaction with follow-up related information and support |
| In the last month the way I feel about myself has changed.^2^ | satisfaction with follow-up related information and support |

^1^ Response options: Strongly agree, moderately agree, slightly agree, slightly disagree, moderately disagree, and strongly disagree.

^2^ Response options: Strongly for the better, moderately for the better, slightly for the better, slightly for the worse, moderately for the worse and strongly for the worse.

**Table S2: Baseline characteristics of TOMBOLA participants eligible to be included in psychosocial analysis**

|  | Cytological surveillance | | Initial colposcopy | |
| --- | --- | --- | --- | --- |
|  | N | % | N | % |
| **Total** | 1703 |  | 1696 |  |
| **Age** |  |  |  |  |
| 20-29 | 737 | 43.3 | 727 | 42.9 |
| 30-39 | 450 | 26.4 | 456 | 26.9 |
| 40-49 | 364 | 21.4 | 357 | 21.0 |
| 50-59 | 152 | 8.9 | 156 | 9.2 |
| **Eligible Smear** |  |  |  |  |
| Mild | 457 | 26.8 | 453 | 26.7 |
| BNA | 1246 | 73.2 | 1243 | 73.3 |
| **HPV status** |  |  |  |  |
| Negative | 898 | 59.2 | 887 | 59.0 |
| Positive | 620 | 40.8 | 616 | 41.0 |
| Missing | 185 | - | 193 | - |
| **Previous BNA cytology test** |  |  |  |  |
| No | 1541 | 90.5 | 1542 | 90.9 |
| Yes | 162 | 9.5 | 154 | 9.1 |
| **Currently using pill** |  |  |  |  |
| No | 1089 | 64.2 | 1138 | 67.4 |
| Yes | 606 | 35.8 | 551 | 32.6 |
| Missing | 8 | - | 7 | - |
| **Trial Centre** |  |  |  |  |
| 1 | 550 | 32.3 | 553 | 32.6 |
| 2 | 428 | 25.1 | 421 | 24.8 |
| 3 | 725 | 42.6 | 722 | 42.6 |
| **Carstairs deprivation index of area of residence** |  |  |  |  |
| 1: least deprived | 252 | 14.8 | 225 | 13.3 |
| 2 | 319 | 18.7 | 314 | 18.5 |
| 3 | 260 | 15.3 | 285 | 16.8 |
| 4 | 449 | 26.4 | 451 | 26.6 |
| 5: most deprived | 423 | 24.8 | 421 | 24.8 |
| **Post school education & training** |  |  |  |  |
| None | 452 | 26.7 | 454 | 26.9 |
| Through work with qualification | 338 | 20 | 329 | 19.5 |
| Qualification other than degree | 499 | 29.5 | 427 | 28 |
| University or college degree | 401 | 23.7 | 430 | 25.5 |
| Missing | 13 | - | 11 | - |
| **Employment status** |  |  |  |  |
| Full time paid | 850 | 50.2 | 829 | 49.1 |
| Part time paid | 398 | 23.5 | 391 | 23.2 |
| Student | 146 | 8.6 | 167 | 9.9 |
| Not in paid employment | 300 | 17.7 | 301 | 17.8 |
| Missing | 9 | - | 8 | - |
| **Marital Status** |  |  |  |  |
| Married/living as married | 957 | 57 | 913 | 54.4 |
| Divorce/widowed/separated | 223 | 13.3 | 227 | 13.5 |
| Single | 498 | 29.7 | 538 | 32.1 |
| Missing | 25 | - | 18 | - |
| **Ethnicity** |  |  |  |  |
| White | 1610 | 95.1 | 1618 | 96.1 |
| Other | 83 | 4.9 | 66 | 3.9 |
| Missing | 10 | - | 12 | - |
| **Reproductive history** |  |  |  |  |
| Never pregnant | 573 | 34.2 | 558 | 33.3 |
| Pregnant, no children | 164 | 9.8 | 197 | 11.7 |
| Pregnant with children | 939 | 56 | 923 | 55 |
| Missing | 27 | - | 18 | - |
| **Smoking status** |  |  |  |  |
| Never smoked | 804 | 47.9 | 804 | 47.8 |
| Ex-smoker | 289 | 17.2 | 287 | 17.1 |
| Current smoker | 587 | 34.9 | 590 | 35.1 |
| Missing | 23 | - | 15 | - |
| **Physical activity** |  |  |  |  |
| Less than once per week | 664 | 3906 | 667 | 39.9 |
| 1-3 times per week | 410 | 24.5 | 378 | 22.6 |
| More than 3 times per week | 601 | 35.9 | 627 | 37.5 |
| Missing | 28 | - | 24 | - |
| **HADS depression at baseline** |  |  |  |  |
| No | 1546 | 90.8 | 1570 | 92.6 |
| Yes | 157 | 9.2 | 126 | 7.4 |
| **HADS anxiety at baseline** |  |  |  |  |
| No | 998 | 58.6 | 1012 | 59.7 |
| Yes | 705 | 41.4 | 684 | 40.3 |
| **Worries score (mean, (SD))** | 1591 | 60.7 (18.6) | 1607 | 60.1 (18.9) |
| Missing N | 112 |  | 89 |  |
| **Satisfaction with information and support score (mean, (SD))** | 1608 | 33.2 (15.7) | 1591 | 33.3 (15.2) |
| Missing N | 95 |  | 105 |  |

**Table S3: Mixed effects model results for the differences between colposcopy and cytology across all time points: secondary analysis (stratified by post-school education)**

|  | **Colposcopy minus cytology^#^** | | |
| --- | --- | --- | --- |
|  | **Estimate** | **95% CI** | **p value** |
|  |  |  |  |
| **Follow-up related worries** |  |  |  |
| No post-school education (N = 827) | -2.21 | (-4.62, 0.19) | 0.071 |
| Qualifications through work (N = 641) | -4.39 | (-7.21, -1.56) | 0.002 |
| Qualification other than degree from college/university (N = 922) | -0.96 | (-3.04, 1.11) | 0.363 |
| Degree (N = 798) | -2.36 | (-4.51, -0.21) | 0.031 |
|  |  |  |  |
| **Satisfaction with follow-up information and support** |  |  |  |
| No post-school education (N = 838) | -2.57 | (-4.34, -0.80) | 0.005 |
| Qualifications through work (N = 633) | -2.80 | (-5.07, -0.53) | 0.016 |
| Qualification other than degree from college/university (N = 920) | -1.51 | (-3.36, 0.35) | 0.112 |
| Degree (N = 798) | -2.25 | (-4.19, -0.32) | 0.023 |

^#^ All models adjusted for age group, eligible smear, HPV status, trial centre, baseline depression and baseline score. N = 10 had missing post-school education
